# *N*,*N*′-Bis[(*E*)-(3-methyl-2-thienyl)methyl­idene]ethane-1,2-diamine

**DOI:** 10.1107/S1600536810041632

**Published:** 2010-10-23

**Authors:** R. Prasath, P. Bhavana, Seik Weng Ng, Edward R. T. Tiekink

**Affiliations:** a Chemistry Group, BITS, Pilani – K. K. Birla Goa Campus, Goa, India 403 726; b Department of Chemistry, University of Malaya, 50603 Kuala Lumpur, Malaysia

## Abstract

Two independent half-mol­ecules, each being completed by inversion symmetry, comprise the asymmetric unit of the title compound, C_14_H_16_N_2_S_2_. The major difference between the mol­ecules is found in the central C—C bond [the C—N—C—C torsion angles are 114.66 (18) and 128.94 (18)° in the two mol­ecules]. The thio­phene and imine groups are almost co-planar in each case [S—C—C—N torsion angles = −6.9 (2) and −3.6 (2)°]. In the crystal, the mol­ecules aggregate into supra­molecular chains *via* C—H⋯π inter­actions.

## Related literature

For background to 2-substituted thio­phenes, see: Campaigne (1984 ▶); Kleemann *et al.* (2006 ▶). For related structures, see: Wang *et al.* (2007 ▶); Wardell *et al.* (2010 ▶).
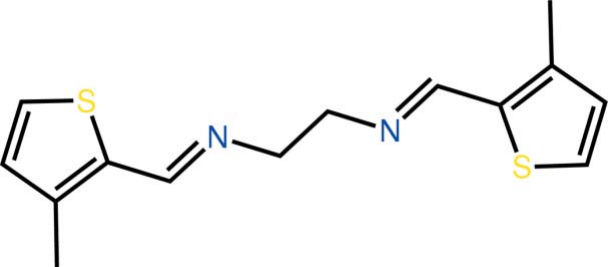


## Experimental

### 

#### Crystal data

C_14_H_16_N_2_S_2_
*M* *_r_* = 276.41
Triclinic, 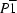

*a* = 8.7610 (6) Å
*b* = 8.9502 (6) Å
*c* = 8.9853 (6) Å
α = 92.760 (1)°
β = 91.653 (1)°
γ = 106.066 (1)°
*V* = 675.61 (8) Å^3^
*Z* = 2
Mo *K*α radiation
μ = 0.38 mm^−1^
*T* = 100 K
0.30 × 0.30 × 0.10 mm

#### Data collection

Bruker SMART APEX diffractometer
Absorption correction: multi-scan (*SADABS*; Sheldrick, 1996 ▶) *T*_min_ = 0.797, *T*_max_ = 0.862
6435 measured reflections
3089 independent reflections
2822 reflections with *I* > 2σ(*I*)
*R*_int_ = 0.019

#### Refinement

*R*[*F* ^2^ > 2σ(*F* ^2^)] = 0.031
*wR*(*F* ^2^) = 0.100
*S* = 1.10
3089 reflections
165 parameters
H-atom parameters constrained
Δρ_max_ = 0.45 e Å^−3^
Δρ_min_ = −0.27 e Å^−3^

### 

Data collection: *APEX2* (Bruker, 2008 ▶); cell refinement: *SAINT* (Bruker, 2008 ▶); data reduction: *SAINT*; program(s) used to solve structure: *SHELXS97* (Sheldrick, 2008 ▶); program(s) used to refine structure: *SHELXL97* (Sheldrick, 2008 ▶); molecular graphics: *ORTEP-3* (Farrugia, 1997 ▶) and *DIAMOND* (Brandenburg, 2006 ▶); software used to prepare material for publication: *publCIF* (Westrip, 2010 ▶).

## Supplementary Material

Crystal structure: contains datablocks general, I. DOI: 10.1107/S1600536810041632/pk2279sup1.cif

Structure factors: contains datablocks I. DOI: 10.1107/S1600536810041632/pk2279Isup2.hkl

Additional supplementary materials:  crystallographic information; 3D view; checkCIF report

## Figures and Tables

**Table 1:** Hydrogen-bond geometry (Å, °) *Cg*1 is the centroid of the S1,C3–C6 ring.

*D*—H⋯*A*	*D*—H	H⋯*A*	*D*⋯*A*	*D*—H⋯*A*
C13—H13⋯*Cg*1^i^	0.95	2.89	3.6179 (19)	134

Symmetry code: (i) 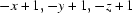.

**Fig. 1.**
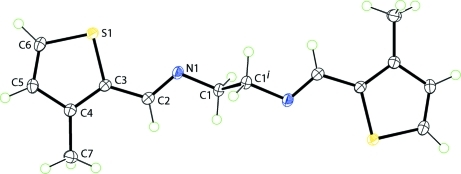
Molecular structure of the first independent molecule showing the atom-labelling scheme and displacement ellipsoids at the 50% probability level. Symmetry operation i: 1 - x, 2 - y, -z.

**Fig. 2.**
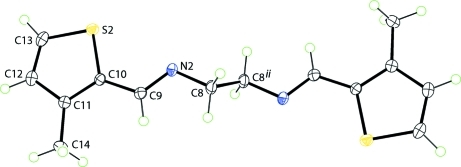
Molecular structure of the second independent molecule in showing the atom-labelling scheme and displacement ellipsoids at the 50% probability level. Symmetry operation ii: -x, 2 - y, 2 - z.

**Fig. 3.**
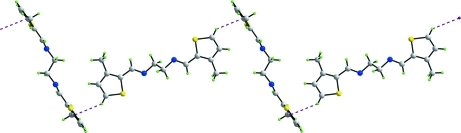
Supramolecular chain sustained by C—H···π contacts (shown as purple dashed lines).

**Fig. 4.**
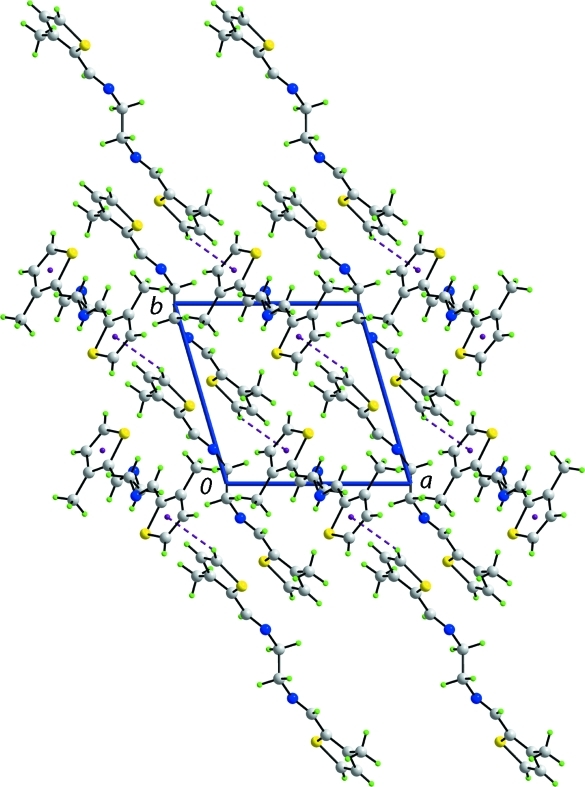
Unit-cell contents viewed in projection along the c axis. The C—H···π contacts are shown as purple dashed lines.

## supplementary crystallographic information

### Comment

The title bifunctional Schiff base features 2-substituted thiophene rings (Campaigne, 1984; Kleemann *et al.*, 2006) of interest owing to their putative biological activity (Wardell *et al.*, 2010). The asymmetric unit comprises two half molecules of, as each of the two independent molecules, Figs 1 and 2, is located about crystallographic centres of inversion. The major difference between the molecules is found around the central C—C bond as manifested in the C2—N1—C1—C1^i^ and C9—N2—C8—C8^ii^ torsion angles of 114.66 (18)° and 128.94 (18)°, respectively [symmetry operation *i*: 1 - *x*, 2 - *y*, -*z* and *ii*: -*x*, 2 - *y*, 2 - *z*]. Further, the twist of the thiophene ring from the imine bond is more pronounced for the first independent molecule [the N1—C2—C3—S1 torsion angle = -6.9 (2)°] compared to that found in the second independent molecule [N2—C9—C10—S2 = -3.6 (2)°]. The conformation about each of the imine N1—C2 [1.274 (2) Å] and N2—C9 [1.272 (2) Å] bonds is *E*. The observed conformations match closely that found for the unsubstituted parent compound (Wang *et al.*, 2007).

The most prominent feature of the crystal packing is the formation of supramolecular chains comprising both independent molecules, Fig. 3, that are sustained by C—H···π interactions, Table 1. The chains pack in layers parallel to (1 1 0), Fig. 4.

### Experimental

A mixture of 3-methyl-2-thiophenecarboxaldehyde (0.43 ml, 0.004 *M*) and ethylenediamine (0.13 ml, 0.002 *M*) was stirred in dichloromethane for 3 h at room temperature. The solvent was removed under reduced pressure, and the resulting solid was dried and purified by column chromatography using a 1:3 mixture of ethyl acetate and hexane. Recrystallization was by slow evaporation of a dichloromethane solution which yielded colourless needles; yield: 81%. *M*. pt. 385–387 K.

### Refinement

Carbon-bound H-atoms were placed in calculated positions (C—H 0.95 to 0.99 Å) and were included in the refinement in the riding model approximation, with *U*_iso_(H) set to 1.2 to 1.5*U*_equiv_(C).

### Crystal data

**Table:**

C_14_H_16_N_2_S_2_	*Z* = 2
*M**_r_* = 276.41	*F*(000) = 292
Triclinic, *P*1	*D*_x_ = 1.359 Mg m^−^^3^
Hall symbol: -P 1	Mo *K*α radiation, λ = 0.71073 Å
*a* = 8.7610 (6) Å	Cell parameters from 4410 reflections
*b* = 8.9502 (6) Å	θ = 4.3–28.3°
*c* = 8.9853 (6) Å	µ = 0.38 mm^−^^1^
α = 92.760 (1)°	*T* = 100 K
β = 91.653 (1)°	Prism, colourless
γ = 106.066 (1)°	0.30 × 0.30 × 0.10 mm
*V* = 675.61 (8) Å^3^	

### Data collection

**Table:**

Bruker SMART APEX diffractometer	3089 independent reflections
Radiation source: fine-focus sealed tube	2822 reflections with *I* > 2σ(*I*)
graphite	*R*_int_ = 0.019
ω scans	θ_max_ = 27.5°, θ_min_ = 2.3°
Absorption correction: multi-scan (*SADABS*; Sheldrick, 1996)	*h* = −11→11
*T*_min_ = 0.797, *T*_max_ = 0.862	*k* = −11→10
6435 measured reflections	*l* = −11→11

### Refinement

**Table:**

Refinement on *F*^2^	Primary atom site location: structure-invariant direct methods
Least-squares matrix: full	Secondary atom site location: difference Fourier map
*R*[*F*^2^ > 2σ(*F*^2^)] = 0.031	Hydrogen site location: inferred from neighbouring sites
*wR*(*F*^2^) = 0.100	H-atom parameters constrained
*S* = 1.10	*w* = 1/[σ^2^(*F*_o_^2^) + (0.0508*P*)^2^ + 0.4238*P*] where *P* = (*F*_o_^2^ + 2*F*_c_^2^)/3
3089 reflections	(Δ/σ)_max_ < 0.001
165 parameters	Δρ_max_ = 0.45 e Å^−^^3^
0 restraints	Δρ_min_ = −0.27 e Å^−^^3^

### Special details

**Table:**

Geometry. All s.u.'s (except the s.u. in the dihedral angle between two l.s. planes) are estimated using the full covariance matrix. The cell s.u.'s are taken into account individually in the estimation of s.u.'s in distances, angles and torsion angles; correlations between s.u.'s in cell parameters are only used when they are defined by crystal symmetry. An approximate (isotropic) treatment of cell s.u.'s is used for estimating s.u.'s involving l.s. planes.
Refinement. Refinement of *F*^2^ against ALL reflections. The weighted *R*-factor *wR* and goodness of fit *S* are based on *F*^2^, conventional *R*-factors *R* are based on *F*, with *F* set to zero for negative *F*^2^. The threshold expression of *F*^2^ > 2σ(*F*^2^) is used only for calculating *R*-factors(gt) *etc*. and is not relevant to the choice of reflections for refinement. *R*-factors based on *F*^2^ are statistically about twice as large as those based on *F*, and *R*- factors based on ALL data will be even larger.

### Fractional atomic coordinates and isotropic or equivalent isotropic displacement parameters (Å^2^)

**Table:**

	*x*	*y*	*z*	*U*_iso_*/*U*_eq_	
S1	0.47805 (4)	0.72178 (4)	0.45069 (4)	0.01583 (12)	
N1	0.48699 (16)	0.93243 (16)	0.19349 (14)	0.0171 (3)	
C1	0.47949 (19)	1.03494 (19)	0.07326 (17)	0.0170 (3)	
H1A	0.5554	1.1387	0.0968	0.020*	
H1B	0.3712	1.0483	0.0637	0.020*	
C2	0.59455 (18)	0.98652 (18)	0.29593 (17)	0.0151 (3)	
H2	0.6641	1.0881	0.2890	0.018*	
C3	0.61453 (18)	0.89825 (18)	0.42300 (17)	0.0147 (3)	
C4	0.73245 (18)	0.93799 (18)	0.53428 (17)	0.0157 (3)	
C5	0.7128 (2)	0.82044 (19)	0.63988 (17)	0.0174 (3)	
H5	0.7844	0.8264	0.7228	0.021*	
C6	0.5808 (2)	0.69924 (19)	0.60906 (17)	0.0180 (3)	
H6	0.5493	0.6120	0.6686	0.022*	
C7	0.8608 (2)	1.0898 (2)	0.5493 (2)	0.0223 (3)	
H7A	0.8213	1.1682	0.6035	0.033*	
H7B	0.8913	1.1247	0.4500	0.033*	
H7C	0.9535	1.0756	0.6043	0.033*	
S2	0.08033 (5)	0.56857 (5)	0.69562 (4)	0.01757 (12)	
N2	0.01928 (16)	0.80946 (15)	0.92178 (15)	0.0166 (3)	
C8	−0.0229 (2)	0.91842 (18)	1.02823 (18)	0.0179 (3)	
H8A	0.0328	0.9189	1.1257	0.021*	
H8B	−0.1388	0.8846	1.0427	0.021*	
C9	0.09982 (18)	0.72358 (18)	0.97335 (17)	0.0151 (3)	
H9	0.1320	0.7369	1.0761	0.018*	
C10	0.14457 (18)	0.60595 (17)	0.88127 (17)	0.0138 (3)	
C11	0.23606 (18)	0.51136 (18)	0.92386 (17)	0.0147 (3)	
C12	0.25375 (19)	0.40900 (18)	0.80306 (18)	0.0177 (3)	
H12	0.3134	0.3356	0.8116	0.021*	
C13	0.1767 (2)	0.42700 (19)	0.67441 (19)	0.0196 (3)	
H13	0.1764	0.3680	0.5836	0.023*	
C14	0.30760 (19)	0.5131 (2)	1.07776 (18)	0.0191 (3)	
H14A	0.3663	0.6200	1.1108	0.029*	
H14B	0.3803	0.4473	1.0766	0.029*	
H14C	0.2229	0.4731	1.1464	0.029*	

### Atomic displacement parameters (Å^2^)

**Table:**

	*U*^11^	*U*^22^	*U*^33^	*U*^12^	*U*^13^	*U*^23^
S1	0.0184 (2)	0.01340 (19)	0.0147 (2)	0.00282 (14)	0.00089 (14)	0.00138 (14)
N1	0.0216 (7)	0.0169 (6)	0.0123 (6)	0.0044 (5)	0.0017 (5)	0.0028 (5)
C1	0.0196 (7)	0.0176 (7)	0.0136 (7)	0.0046 (6)	0.0003 (6)	0.0034 (6)
C2	0.0176 (7)	0.0133 (7)	0.0152 (7)	0.0054 (6)	0.0039 (6)	0.0008 (5)
C3	0.0172 (7)	0.0130 (7)	0.0142 (7)	0.0045 (6)	0.0029 (6)	0.0009 (5)
C4	0.0185 (7)	0.0146 (7)	0.0149 (7)	0.0061 (6)	0.0014 (6)	0.0001 (6)
C5	0.0229 (8)	0.0178 (7)	0.0133 (7)	0.0090 (6)	−0.0003 (6)	0.0006 (6)
C6	0.0250 (8)	0.0171 (7)	0.0140 (7)	0.0087 (6)	0.0034 (6)	0.0031 (6)
C7	0.0213 (8)	0.0178 (8)	0.0256 (8)	0.0024 (6)	−0.0047 (6)	0.0022 (6)
S2	0.0210 (2)	0.0169 (2)	0.0142 (2)	0.00442 (15)	−0.00034 (14)	0.00084 (14)
N2	0.0193 (6)	0.0113 (6)	0.0188 (6)	0.0040 (5)	−0.0001 (5)	−0.0004 (5)
C8	0.0230 (8)	0.0132 (8)	0.0187 (7)	0.0068 (6)	0.0021 (6)	0.0007 (6)
C9	0.0148 (7)	0.0129 (7)	0.0159 (7)	0.0010 (5)	0.0007 (5)	0.0015 (6)
C10	0.0148 (7)	0.0115 (7)	0.0144 (7)	0.0022 (5)	0.0018 (5)	0.0012 (5)
C11	0.0134 (7)	0.0138 (7)	0.0160 (7)	0.0017 (5)	0.0032 (5)	0.0024 (6)
C12	0.0163 (7)	0.0141 (7)	0.0225 (8)	0.0034 (6)	0.0059 (6)	0.0011 (6)
C13	0.0214 (8)	0.0158 (7)	0.0199 (8)	0.0026 (6)	0.0061 (6)	−0.0026 (6)
C14	0.0171 (7)	0.0228 (8)	0.0186 (8)	0.0069 (6)	−0.0005 (6)	0.0034 (6)

### Geometric parameters (Å, °)

**Table:**

S1—C6	1.7119 (16)	S2—C13	1.7125 (17)
S1—C3	1.7309 (16)	S2—C10	1.7318 (16)
N1—C2	1.274 (2)	N2—C9	1.272 (2)
N1—C1	1.4621 (19)	N2—C8	1.460 (2)
C1—C1^i^	1.525 (3)	C8—C8^ii^	1.520 (3)
C1—H1A	0.9900	C8—H8A	0.9900
C1—H1B	0.9900	C8—H8B	0.9900
C2—C3	1.453 (2)	C9—C10	1.453 (2)
C2—H2	0.9500	C9—H9	0.9500
C3—C4	1.377 (2)	C10—C11	1.377 (2)
C4—C5	1.429 (2)	C11—C12	1.426 (2)
C4—C7	1.502 (2)	C11—C14	1.499 (2)
C5—C6	1.360 (2)	C12—C13	1.361 (2)
C5—H5	0.9500	C12—H12	0.9500
C6—H6	0.9500	C13—H13	0.9500
C7—H7A	0.9800	C14—H14A	0.9800
C7—H7B	0.9800	C14—H14B	0.9800
C7—H7C	0.9800	C14—H14C	0.9800
			
C6—S1—C3	91.47 (8)	C13—S2—C10	91.55 (8)
C2—N1—C1	116.35 (13)	C9—N2—C8	116.78 (14)
N1—C1—C1^i^	109.83 (16)	N2—C8—C8^ii^	110.34 (17)
N1—C1—H1A	109.7	N2—C8—H8A	109.6
C1^i^—C1—H1A	109.7	C8^ii^—C8—H8A	109.6
N1—C1—H1B	109.7	N2—C8—H8B	109.6
C1^i^—C1—H1B	109.7	C8^ii^—C8—H8B	109.6
H1A—C1—H1B	108.2	H8A—C8—H8B	108.1
N1—C2—C3	122.28 (14)	N2—C9—C10	122.61 (14)
N1—C2—H2	118.9	N2—C9—H9	118.7
C3—C2—H2	118.9	C10—C9—H9	118.7
C4—C3—C2	127.93 (14)	C11—C10—C9	127.42 (14)
C4—C3—S1	111.67 (12)	C11—C10—S2	111.68 (12)
C2—C3—S1	120.39 (12)	C9—C10—S2	120.89 (11)
C3—C4—C5	111.63 (14)	C10—C11—C12	111.52 (14)
C3—C4—C7	124.67 (14)	C10—C11—C14	124.84 (14)
C5—C4—C7	123.60 (14)	C12—C11—C14	123.64 (14)
C6—C5—C4	112.87 (14)	C13—C12—C11	113.24 (14)
C6—C5—H5	123.6	C13—C12—H12	123.4
C4—C5—H5	123.6	C11—C12—H12	123.4
C5—C6—S1	112.33 (12)	C12—C13—S2	112.00 (12)
C5—C6—H6	123.8	C12—C13—H13	124.0
S1—C6—H6	123.8	S2—C13—H13	124.0
C4—C7—H7A	109.5	C11—C14—H14A	109.5
C4—C7—H7B	109.5	C11—C14—H14B	109.5
H7A—C7—H7B	109.5	H14A—C14—H14B	109.5
C4—C7—H7C	109.5	C11—C14—H14C	109.5
H7A—C7—H7C	109.5	H14A—C14—H14C	109.5
H7B—C7—H7C	109.5	H14B—C14—H14C	109.5
			
C2—N1—C1—C1^i^	114.66 (18)	C9—N2—C8—C8^ii^	128.94 (18)
C1—N1—C2—C3	179.26 (14)	C8—N2—C9—C10	177.52 (14)
N1—C2—C3—C4	174.57 (16)	N2—C9—C10—C11	177.29 (16)
N1—C2—C3—S1	−6.9 (2)	N2—C9—C10—S2	−3.6 (2)
C6—S1—C3—C4	−1.13 (13)	C13—S2—C10—C11	−0.31 (12)
C6—S1—C3—C2	−179.90 (13)	C13—S2—C10—C9	−179.53 (13)
C2—C3—C4—C5	−179.55 (15)	C9—C10—C11—C12	179.52 (15)
S1—C3—C4—C5	1.80 (17)	S2—C10—C11—C12	0.36 (17)
C2—C3—C4—C7	3.9 (3)	C9—C10—C11—C14	0.1 (3)
S1—C3—C4—C7	−174.74 (13)	S2—C10—C11—C14	−179.05 (12)
C3—C4—C5—C6	−1.7 (2)	C10—C11—C12—C13	−0.2 (2)
C7—C4—C5—C6	174.86 (15)	C14—C11—C12—C13	179.18 (14)
C4—C5—C6—S1	0.86 (18)	C11—C12—C13—S2	0.01 (18)
C3—S1—C6—C5	0.14 (13)	C10—S2—C13—C12	0.17 (13)

Symmetry codes: (i) −*x*+1, −*y*+2, −*z*; (ii) −*x*, −*y*+2, −*z*+2.

### Hydrogen-bond geometry (Å, °)

**Table:**

Cg1 is the centroid of the S1,C3–C6 ring.

**Table:**

*D*—H···*A*	*D*—H	H···*A*	*D*···*A*	*D*—H···*A*
C13—H13···Cg1^iii^	0.95	2.89	3.6179 (19)	134

Symmetry codes: (iii) −*x*+1, −*y*+1, −*z*+1.
